# Axial Length Distributions in Patients With Genetically Confirmed Inherited Retinal Diseases

**DOI:** 10.1167/iovs.63.6.15

**Published:** 2022-06-15

**Authors:** Katie M. Williams, Michalis Georgiou, Angelos Kalitzeos, Isabelle Chow, Pirro G. Hysi, Anthony G. Robson, Gareth Lingham, Fred K. Chen, David A. Mackey, Andrew R. Webster, Christopher J. Hammond, Polina Prokhoda, Joseph Carroll, Michel Michaelides, Omar A. Mahroo

**Affiliations:** 1 UCL Institute of Ophthalmology, University College London, London, United Kingdom; 2 Moorfields Eye Hospital, London, United Kingdom; 3 Section of Academic Ophthalmology, School of Life Course Sciences, FoLSM, King's College London, United Kingdom; 4 Department of Twin Research and Genetic Epidemiology, School of Life Course Sciences, FoLSM, King's College London, London, United Kingdom; 5 Jones Eye Institute, Department of Ophthalmology, University of Arkansas for Medical Sciences, Little Rock, Arkansas, United States; 6 Centre for Ophthalmology and Visual Sciences (Incorporating Lions Eye Institute), The University of Western Australia, Perth, Western Australia, Australia; 7 Ophthalmology, Department of Surgery, University of Melbourne, Melbourne, Victoria, Australia; 8 Department of Ophthalmology and Visual Sciences, Medical College of Wisconsin, Milwaukee, Wisconsin, United States; 9 Department of Cell Biology, Neurobiology and Anatomy, Medical College of Wisconsin, Milwaukee, Wisconsin, United States

**Keywords:** retina, rod–cone dystrophy, cone–rod dystrophy, macular dystrophy, albinism, eye axial length, myopia, hyperopia

## Abstract

**Purpose:**

We investigated axial length (AL) distributions in inherited retinal diseases (IRDs), comparing them with reference cohorts.

**Methods:**

AL measurements from IRD natural history study participants were included and compared with reference cohorts (TwinsUK, Raine Study Gen2-20, and published studies). Comparing with the Raine Study cohort, formal odds ratios (ORs) for AL ≥ 26 mm or AL ≤ 22 mm were derived for each IRD (Firth's logistic regression model, adjusted for age and sex).

**Results:**

Measurements were available for 435 patients (median age, 19.5 years). Of 19 diseases, 10 had >10 participants: *ABCA4* retinopathy; *CNGB3-* and *CNGA3*-associated achromatopsia; *RPGR*-associated disease; *RPE65*-associated disease; blue cone monochromacy (BCM); Bornholm eye disease (BED); *TYR*- and *OCA2*-associated oculocutaneous albinism; and *GPR143*-associated ocular albinism. Compared with the TwinsUK cohort (*n* = 322; median age, 65.1 years) and Raine Study cohort (*n* = 1335; median age, 19.9 years), AL distributions were wider in the IRD groups. Increased odds for longer ALs were observed for BCM, BED, *RPGR*, *RPE65*, *OCA2*, and *TYR*; increased odds for short AL were observed for *RPE65*, *TYR*, and *GPR143*. In subanalysis of *RPGR*-associated disease, longer average ALs occurred in cone–rod dystrophy (*n* = 5) than rod–cone dystrophy (*P* = 0.002).

**Conclusions:**

Several diseases showed increased odds for longer AL (highest OR with BCM); some showed increased odds for shorter AL (highest OR with *GPR143*). Patients with *RPE65*- and *TYR*-associated disease showed increased odds for longer and for shorter eyes. Albinism genes were associated with different effects on AL. These findings add to the phenotype of IRDs and may yield insights into mechanisms of refractive error development.

The prevalence of myopic refractive error is increasing worldwide, and this has significant public health implications.^1^ Retinal signaling pathways appear to be important in driving growth of the eye and hence in the process of emmetropization.^2^^,^^3^ Refractive error is a feature of many inherited retinal diseases (IRDs), but this can vary widely among different conditions.^4^ Refractive error is a function of corneal curvature, lens power, and axial length (AL). However, the most common form of myopia, termed axial myopia, is directly attributable to an abnormally long AL. Research into the genetic architecture of myopia suggests that the myopic signaling cascade directly affects scleral growth.^5^ Therefore, AL measurements, rather than simply the overall refractive error, might allow more direct assessment of scleral growth and factors affecting myopia development.

Given that a number of IRDs selectively attenuate particular retinal signaling pathways, investigating and comparing AL in these conditions could provide insights into the processes driving eye growth and emmetropization. A large number of patients with genetically confirmed IRDs (>400) at two centers (Moorfields Eye Hospital, London, UK,^6^ and Medical College of Wisconsin, Milwaukee, WI, USA) underwent AL measurements using optical biometry as part of previous natural history studies.^7^^–^^12^ In this study, we describe AL distributions in the range of IRDs that were included. We also compare these with a number of reference datasets, including data from the Raine Study Gen2-20 follow-up and TwinsUK cohorts, as well as published data from several international cohorts.

## Methods

### IRD Participants

Patients who had undergone AL measurements as part of prospective research studies were included. The cohort included patients with variants in genes associated with rod–cone dystrophies, Leber congenital amaurosis, Stargardt disease, cone dystrophies, achromatopsia (ACHM), oculocutaneous albinism (OCA), and ocular albinism. These specific genetic conditions were chosen because they have been the subject of natural history or deep phenotyping studies to establish descriptive parameters of disease state or progression that could inform future potential therapeutic trials.^7^^–^^12^ The specific genes are listed in the Results.

### Reference Cohorts

In addition to comparing AL among diseases, we compared ALs with reference cohorts. The Raine Study (formerly known as the Western Australian Pregnancy Cohort) is an ongoing study of a cohort of participants (Gen2) who have participated in regular follow-up since their birth between 1989 and 1992. At the age of 20 years, eligible participants (>2000) were invited to take part in a comprehensive eye assessment (Raine Study Gen2-20-year follow-up), including measurement of AL.^13^ TwinsUK is a UK-wide registry of ∼14,000 largely healthy adult twins who have volunteered to participate in research studies based at St Thomas’ Hospital in London.^14^ In addition, we extracted mean AL values from the published literature of other large healthy cohorts: the Avon Longitudinal Study of Parents and Children (ALSPAC), which measured AL in 2625 participants 15 years old^15^; the Generation R study, which measured AL in its pediatric cohort at the age of 6 years (*n* = 6690) and again at the age of 9 years (*n* = 5862)^16^; and the Rotterdam Study III (RS-III), which is a prospective study of individuals 45 years or older that has measured ALs in 2957 participants.^17^ Finally, we also included mean AL measurements from a large group of patients (*n* = 4571) undergoing cataract surgery at a single center from a previous study.^18^ As the Raine Study participants were found to have an average age comparable to that of the IRD participants, further formal statistical comparisons (described below) were undertaken using this cohort.

### AL Measurement

All participants underwent AL measurement by optical biometry. Participants with IRD and participants from the Raine Study underwent measurement of AL by the ZEISS IOLMaster (Carl Zeiss Meditec, Jena, Germany); TwinsUK participants underwent AL measurement by the Zeiss IOLMaster, the NIDEK AL-Scan (Nidek Co., Ltd., Japan), or the LENSTAR Optical Biometer (Haag-Streit, Koeniz, Switzerland). AL was measured in both eyes. In all cohorts, measurements were made by trained research staff. The average AL between both eyes for each participant was used in our analyses (except for the interocular symmetry analysis).

### Statistical Testing

For the IRD participants, further analysis was performed for those conditions with more than 10 individuals. Firth's logistic regression models were constructed for the binary outcome of an AL ≤ 22 mm and, second, for an AL ≥ 26 mm for each IRD subtype when compared to a reference cohort (Raine Study). Firth's logistic regression has become a standard approach for the analysis of binary outcomes with small samples. The particular cut-offs were chosen as these are frequently used to define short and long eyes, particularly in clinical contexts, such as in the planning of cataract surgery and lens replacement.^19^^–^^25^

Adjusted odds ratios (ORs) were obtained with adjustment for age and sex. For this analysis, only one IRD patient was included per family. For X-linked conditions (where only affected males had been recruited), only males were included from the comparison group. Differences in AL among different electrophysiological groups with *ABCA4*-associated disease (groups 1 to 3),^10^^,^^26^ clinical phenotypes in *RPGR*-associated disease, *CNGA3*- and *CNGB3*-associated ACHM, and among different genes associated with albinism were also examined using simple *t*-tests and linear regression models. Stata 13.1 (StataCorp, College Station, TX, USA) and R (R Foundation for Statistical Computing, Vienna, Austria) were used for the analyses and graphical outputs.

### Ethical Approval

Study procedures had relevant local ethics committee approval as follows: Northwest London (Moorfields IRD patient cohort); Human Research Ethics Committee of the University of Western Australia (Raine Study RA/4/20/5722); National Research Ethics Service Committee London–Harrow (TwinsUK); and Medical College of Wisconsin (PRO17439). Participants gave informed consent, and procedures conformed to the tenets of the Declaration of Helsinki.

## Results

### AL Measurements and Demographics of IRD Participants

AL measurements were available for 435 patients with IRD; median age was 19.5 years (interquartile range [IQR], 13.1–30.7). As expected, because some of the conditions are X-linked, more than half of the IRD group was male (62%). Table 1 gives the full range of conditions, together with median patient age at assessment, sex distribution, and mean ALs. Age of onset was only available for individuals with *ABCA4*-associated disease, where symptom onset occurred at a median of 11 years (IQR, 7–14). The mean ± SD AL for all patients was 24.03 ± 2.00 mm. Taking the broadly normal range for AL as ∼22 to 26 mm,^19^^–^^25^^,^^27^ there were no conditions in this cohort with mean AL below the lower limit of this range, but for four conditions the mean AL was above the upper limit of this range.

**Table 1.** Inherited Retinal Disease Types With Numbers of Participants, Age, Sex, and Mean ALs

		Age at Measurement of AL (y)			AL (mm)	
Inherited Condition	*n*	Median	IQR	Sex (% Male)	Mean	SD
Conditions with >10 participants						
*RPE65*-associated autosomal recessive retinal dystrophy	29	18.0	11.9–21.1	48.3	23.27	2.16
*CNGA3*-associated achromatopsia	36	22.7	13.5–32.5	50.0	23.87	1.72
*CNGB3*-associated achromatopsia	65	18.9	13.3–27.3	50.8	23.15	1.67
*ABCA4*-associated retinopathy (Stargardt disease)	93	16.3	11.5–27.5	43.0	23.67	1.20
*RPGR*-related disease (rod–cone dystrophy, *n* = 71; cone or cone–rod dystrophy, *n* = 5)	76	24.8	15.7–31.7	100	24.73	1.64
Blue cone monochromacy	19	18.0	11.8–34.5	100	26.32	2.01
Bornholm eye disease	20	16.5	13.1–24.7	100	26.59	2.13
*TYR*-associated oculocutaneous albinism	28	21.0	11.8–36.0	42.9	22.60	2.18
*GPR143*-associated ocular albinism	11	19.0	10.2–30.5	100	23.25	1.51
*OCA2*-associated oculocutaneous albinism	18	16.8	13.3–30.6	50.0	23.97	1.80
Other conditions						
*PDE6C*-associated achromatopsia	6	41.0	36.4–46.4	16.7	25.74	2.36
*ATF6*-associated achromatopsia	8	26.3	17.3–42.3	25.0	23.03	2.40
*GNAT2*-associated achromatopsia	6	31.0	10.5–43.8	66.7	24.03	2.97
*KCNV2*-associated retinopathy (cone dystrophy with supernormal rod response)	3	25.7	23.1–67.7	100	26.40	2.98
*R9AP*-associated disease (cone dysfunction with “bradyopsia”)	2	21.7	17.8–25.5	0.0	27.00	2.96
*GUCY2D*-associated disease (autosomal dominant cone or cone–rod dystrophy)	6	46.3	24.7–49.2	33.3	24.67	1.24
*PROM1*-associated disease (autosomal dominant macular or retinal dystrophy)	6	43.0	31.2–49.1	66.7	24.26	1.00
*RDH12*-associated disease (autosomal dominant rod–cone dystrophy)	1	14.2	—	0.0	23.19	—
*IMPG2*-associated disease (autosomal recessive retinal dystrophy)	2	26.0	25.7–26.3	0.0	23.44	1.50
Total	435	19.5	13.1–30.7	61.6	24.03	2.00

### Interocular Symmetry

Pearson correlations between right- and left-eye ALs were high in both IRD and reference datasets: *TYR*, *r* = 0.98 (95% confidence interval [CI], 0.96–0.99); *OCA2*, *r* = 0.97 (95% CI, 0.91–0.99); *GPR143*, *r* = 0.86 (95% CI, 0.54–0.96); *RPE65*, *r* = 0.98 (95% CI, 0.96–0.99); *CNGB3*, *r* = 0.97 (95% CI, 0.95–0.98); *CNGA3*, *r* = 0.99 (95% CI, 0.98–0.99); *ABCA4*, *r* = 0.93 (95% CI, 0.90–0.96); *RPGR*, *r* = 0.98 (95% CI, 0.96–0.98); blue cone monochromacy (BCM), *r* = 0.98 (95% CI, 0.96–0.99); Bornholm eye disease (BED), *r* = 0.98 (95% CI, 0.94–0.99); TwinsUK, *r* = 0.94 (95% CI, 0.93–0.95); and Raine Study, *r* = 0.97 (95% CI, 0.97–0.98). Given the high degree of symmetry, we averaged AL values between eyes in each subject for subsequent analyses.

### Comparison With Healthy Cohorts

**Figure 1.**
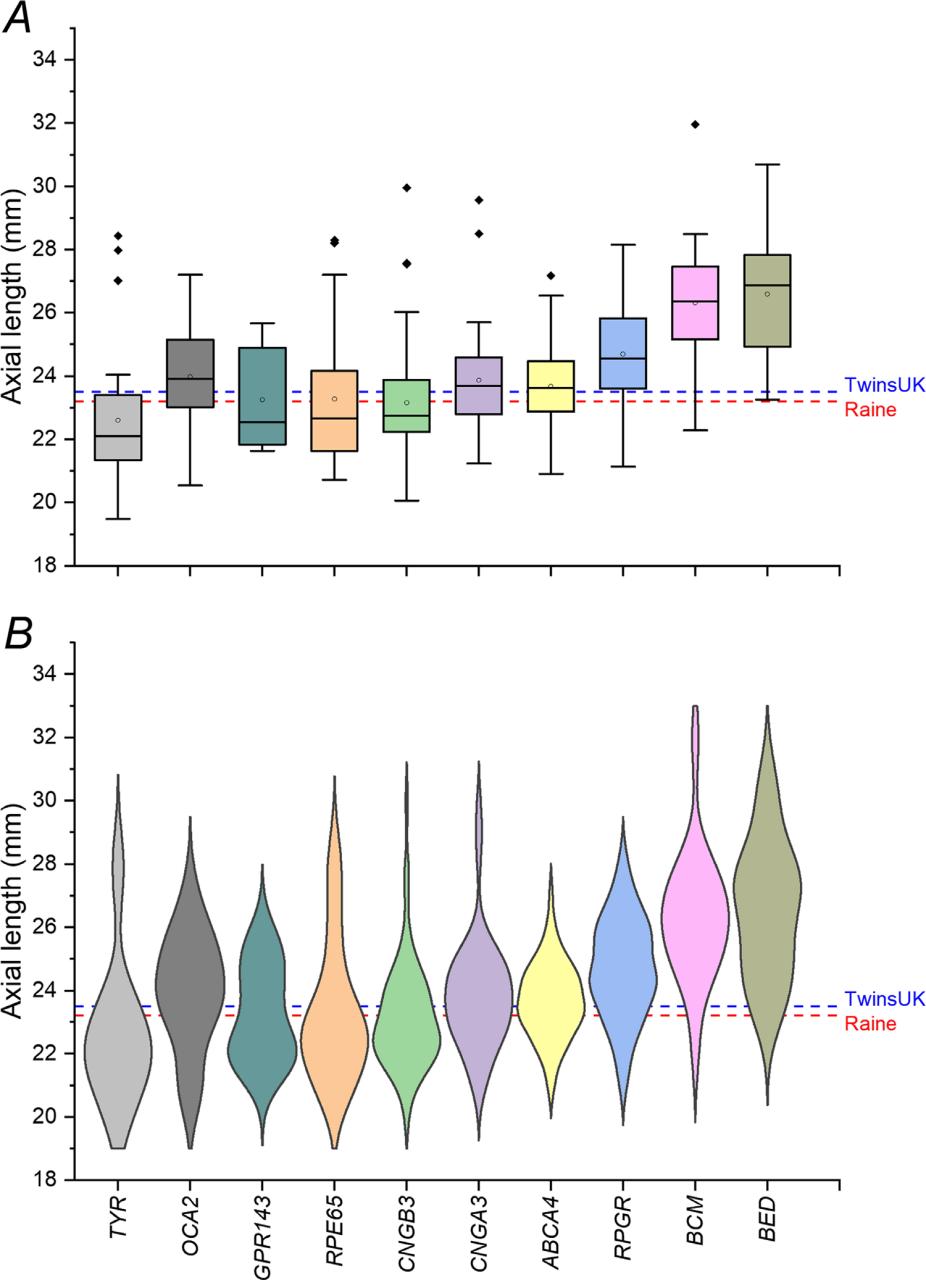
Axial lengths in inherited retinal diseases for which there were more than 10 participants. (**A**) Boxplots (*open circles* denote means; *filled diamonds* denote outliers). (**B**) Violin plots. *Dashed lines* show mean ALs for the TwinsUK and Raine Study cohorts as labeled. Compared with the reference cohorts, average ALs were clearly longer in BCM, BED, and *RPGR*- and *OCA2*-associated disease.

Figure 1 shows ALs for those conditions for which there were more than 10 individuals, as well as the means for two healthy cohorts (TwinsUK and Raine Study). Average AL was longest in BED, BCM, and *RPGR*-associated disease. Interestingly, patients with *OCA2*-associated OCA showed a longer mean AL compared with the reference cohorts, whereas those with *TYR*-associated OCA had a shorter mean AL. The mean AL ± SD for the TwinsUK cohort was 23.31 ± 1.12 mm (322 individuals; mean age ± SD, 63.6 ± 11.0 years). The mean AL ± SD for the Raine Study was 23.60 ± 0.93 mm (1335 unrelated individuals; mean age ± SD, 20.0 ± 0.46 years). The violin plots in the lower panel also show that for some conditions there was a more complex, bimodal distribution.

**Figure 2.**
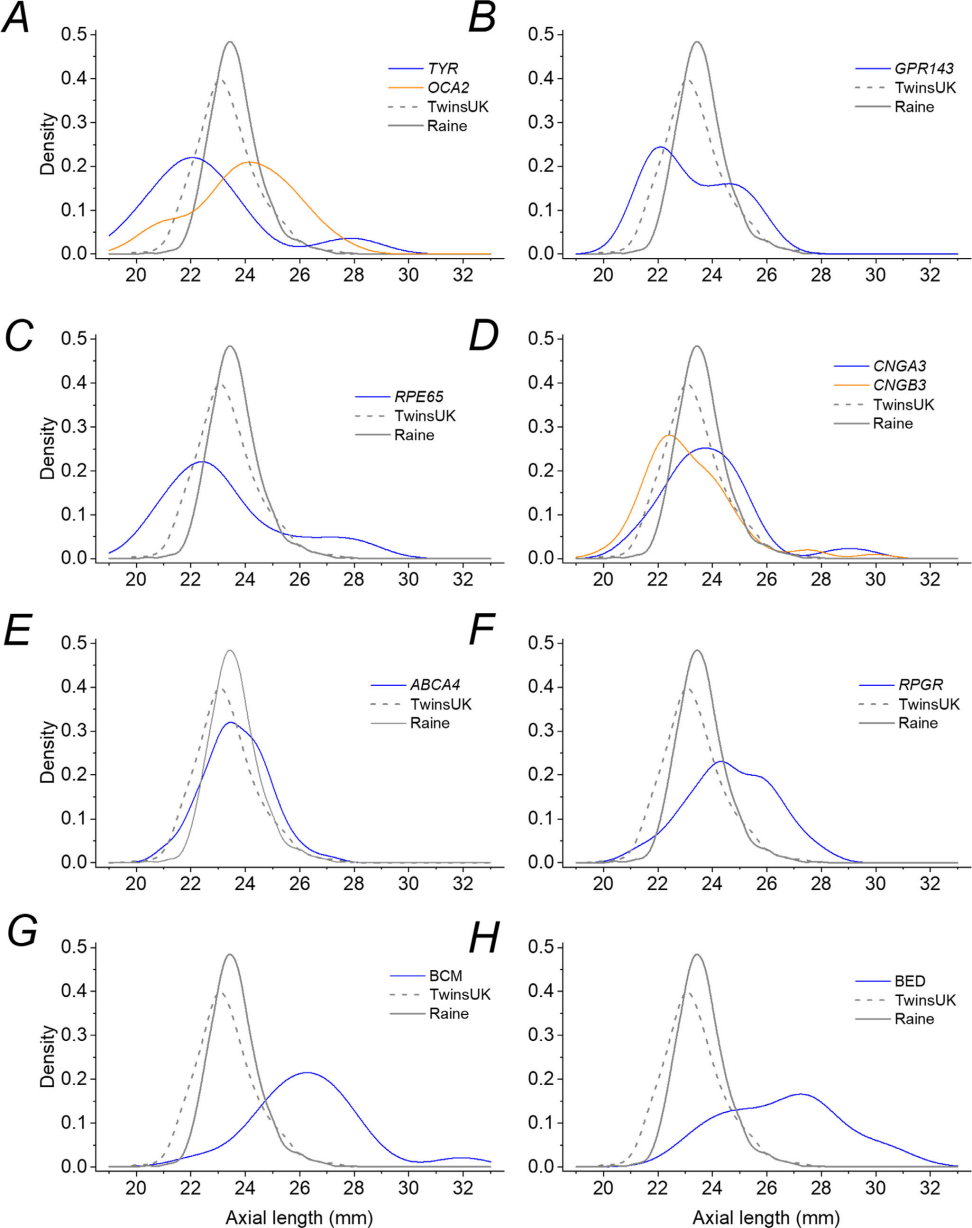
Density distributions for axial lengths for each IRD subtype. *Blue curves* plot the density for each condition in each panel as follows: (**A**) *TYR*-associated albinism (*orange curve* shows the density for *OCA2*-associated albinism); (**B**) *GPR143*-associated ocular albinism; (**C**) *RPE65*-associated disease; (**D**) *CNGA3*-associated achromatopsia (*orange curve* shows the density for *CNGB3*-associated achromatopsia); (**E**) *ABCA4*-associated retinopathy (Stargardt disease); (**F**) *RPGR*-associated disease; (**G**) BCM; and (**H**) BED. The *gray* *solid* and *dashed curves* in each panel show distributions for the Raine Study and TwinsUK cohorts, respectively.

Figure 2 illustrates the distributions more clearly, with density plots for each IRD and plots for the TwinsUK and Raine Study cohorts shown in each panel for comparison. The healthy cohorts show relatively tight distributions, whereas for the IRDs the distributions appear broader, with additional or shifted peaks visible for some conditions. With the exception of *ABCA4*, all IRD groups showed significantly greater variance than both the TwinsUK and Raine Study cohorts (*P* < 0.05, Levene's test for equal variance). For *ABCA4*-associated retinopathy, the variance of AL in the patient group was significantly greater than that for the Raine Study participants (*P* < 0.001), but not significantly different from the TwinsUK cohort (*P* = 0.34).

**Figure 3.**
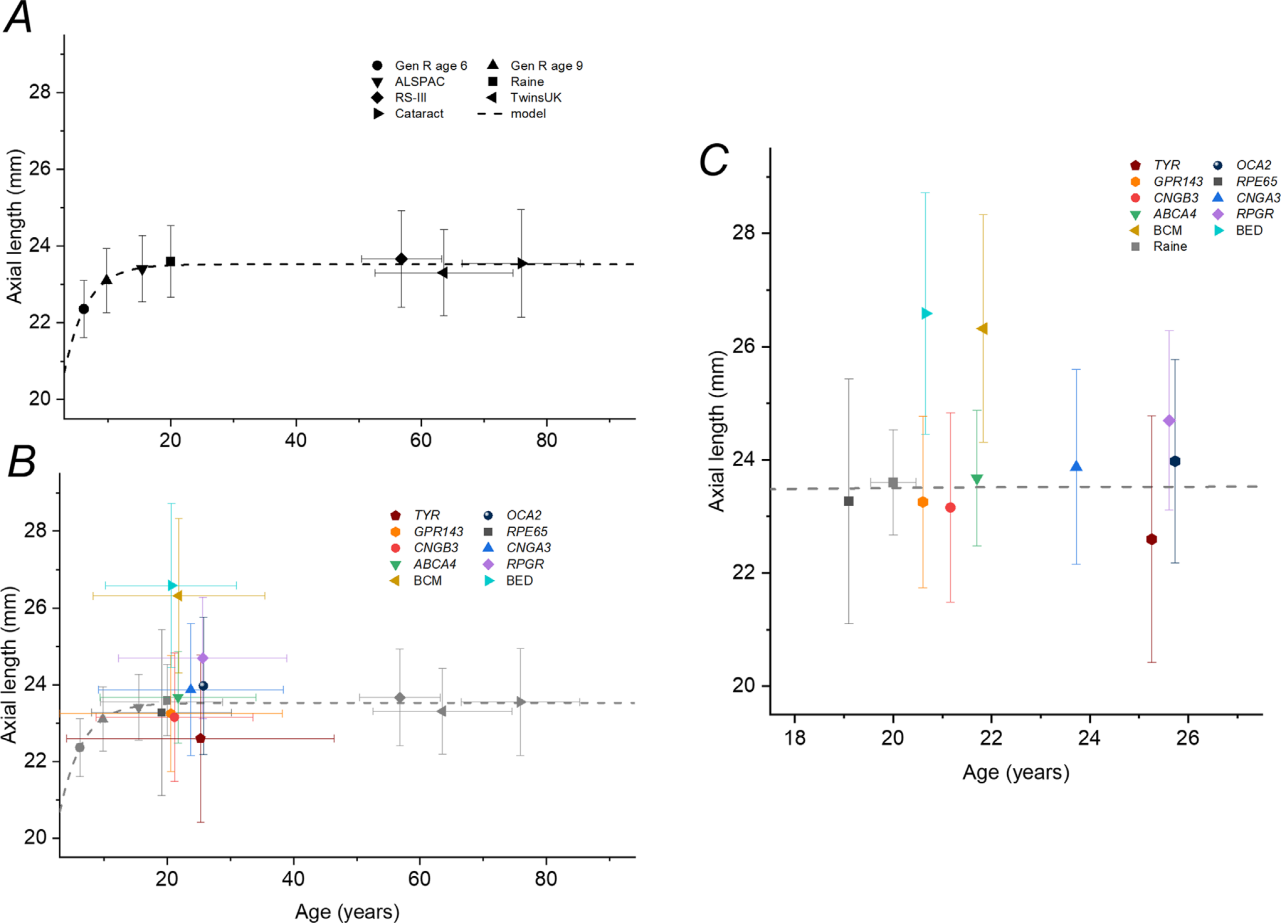
Mean ALs plotted against average age for reference cohorts and IRD patients. (**A**) Mean ALs for a number of reference cohorts of different ages. *Horizontal* and *vertical error bars* show standard deviations. The *dashed curve* plots an exponential recovery relation fitted empirically to the data. Note that, because these are not longitudinal data, there will be a cohort effect (with increasing prevalence of myopia in many populations), so the younger individuals are likely in the future to have longer eyes when they reach the age of the older cohorts in comparison to those cohorts. (**B**) Data for IRD subgroups (*colored symbols*) superimposed on reference data (here shown in *gray*). (**C**) Data from (**B**) are replotted with an expanded *x*-axis covering the mean ages of the patient cohorts and with the horizontal error bars for the patient cohorts removed to aid clarity.

As AL varies with age (in a nonlinear manner), this can potentially confound comparisons. We sought additional published reference cohorts of different ages. Summary data for these are presented in Table 2 (together with data for the TwinsUK and Raine Study cohorts). Figure 3 plots mean ALs against age for the reference cohorts (upper panel), fitted with an empirical expression, to illustrate the relationship in healthy individuals. The lower panel superimposes data from the IRD groups for comparison, and the right-hand panel expands the *x*-axis and removes the horizontal error bars for clarity.

**Table 2.** Mean ALs for Reference Cohorts, Ordered by Increasing Age

		Age (y)		AL (mm)		
Cohort	*n*	Mean	SD	Mean	SD	Range
Generation R	6084	6.17	0.52	22.36	0.75	—
Generation R	5296	9.79	0.33	23.10	0.84	—
ALSPAC	2495	15.47	0.32	23.41	0.86	—
Raine Study	1335	20.0	0.46	23.60	0.93	20.37–27.95
RS-III^10^	2957	56.8	6.4	23.67	1.26	21.82–25.90
TwinsUK	322	63.6	11.0	23.31	1.12	20.06–27.30
Large unselected cataract cohort^18^	4571	75.9	9.4	23.55	1.40	—

**Figure 4.**
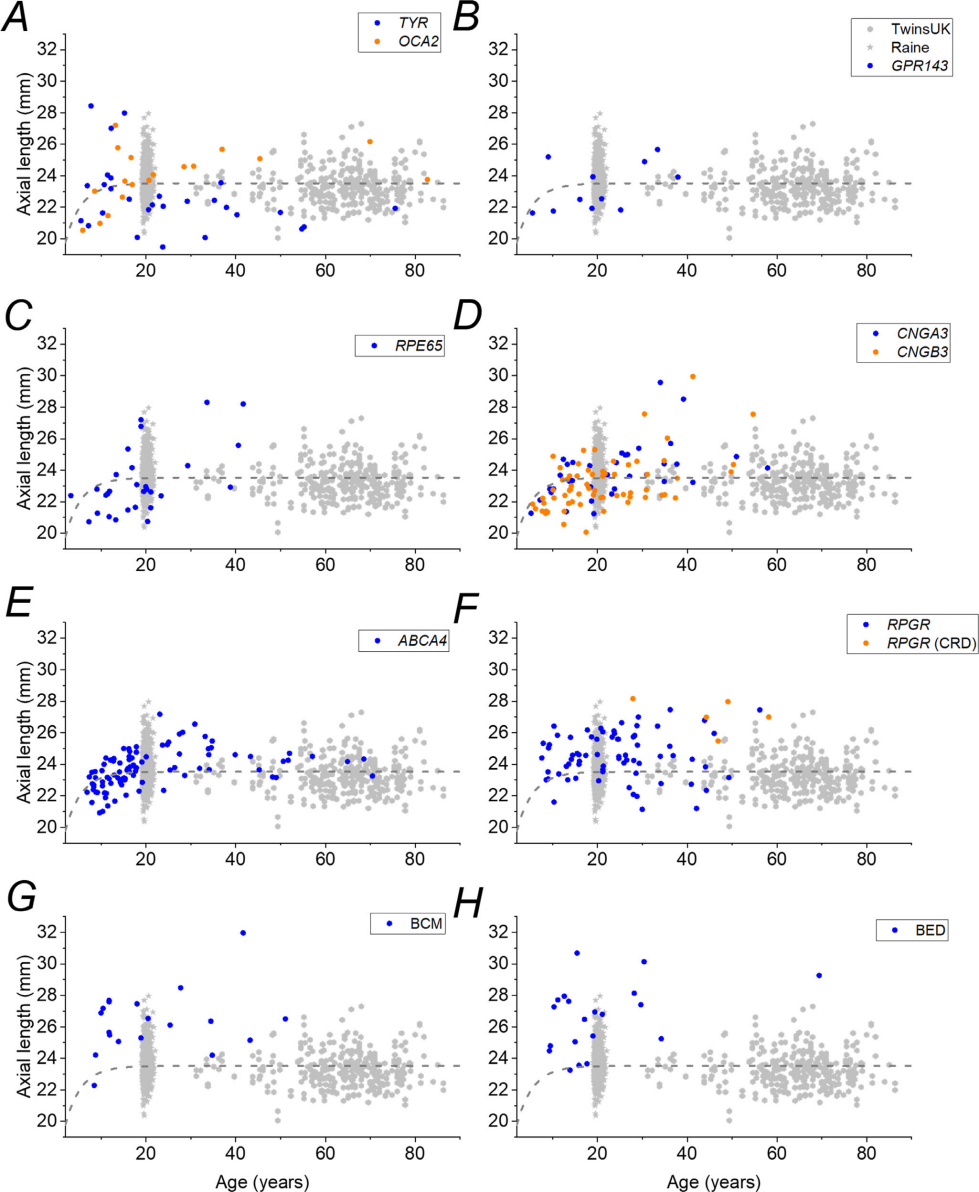
Axial lengths plotted against age for individual participants in each IRD group. *Blue symbols* plot data for IRD patients as follows: (**A**) *TYR*-associated albinism (*orange symbols* for *OCA2*-associated albinism); (**B**) *GPR143*-associated ocular albinism; (**C**) *RPE65*-associated disease; (**D**) *CNGA3*-associated achromatopsia (*orange symbols* for *CNGB3*-associated achromatopsia); (**E**) *ABCA4*-associated retinopathy (Stargardt disease); (**F**) *RPGR*-associated disease (*orange symbols* for cone–rod disease); (**G**) BCM; and (**H**) BED. *Gray* *hexagons* and *stars* in each panel show distributions for the TwinsUK and Raine Study cohorts, respectively. The *dashed curve* in each panel re-plots empirical relations describing reference data in Figure 3.

Figure 4 plots AL against age by individual patient for each IRD to show the data with more granularity. The gray symbols plot the individual measurements for the TwinsUK and Raine Study participants for comparison, and the empirical relation (dashed curve from Fig. 3) describing the reference cohorts is also replotted. The majority of individuals fall above the reference curve (longer AL) for *RPGR*, BCM, and BED, but the majority of individuals fall below the reference line for *RPE65*- and *TYR*-associated disease.

**Figure 5.**
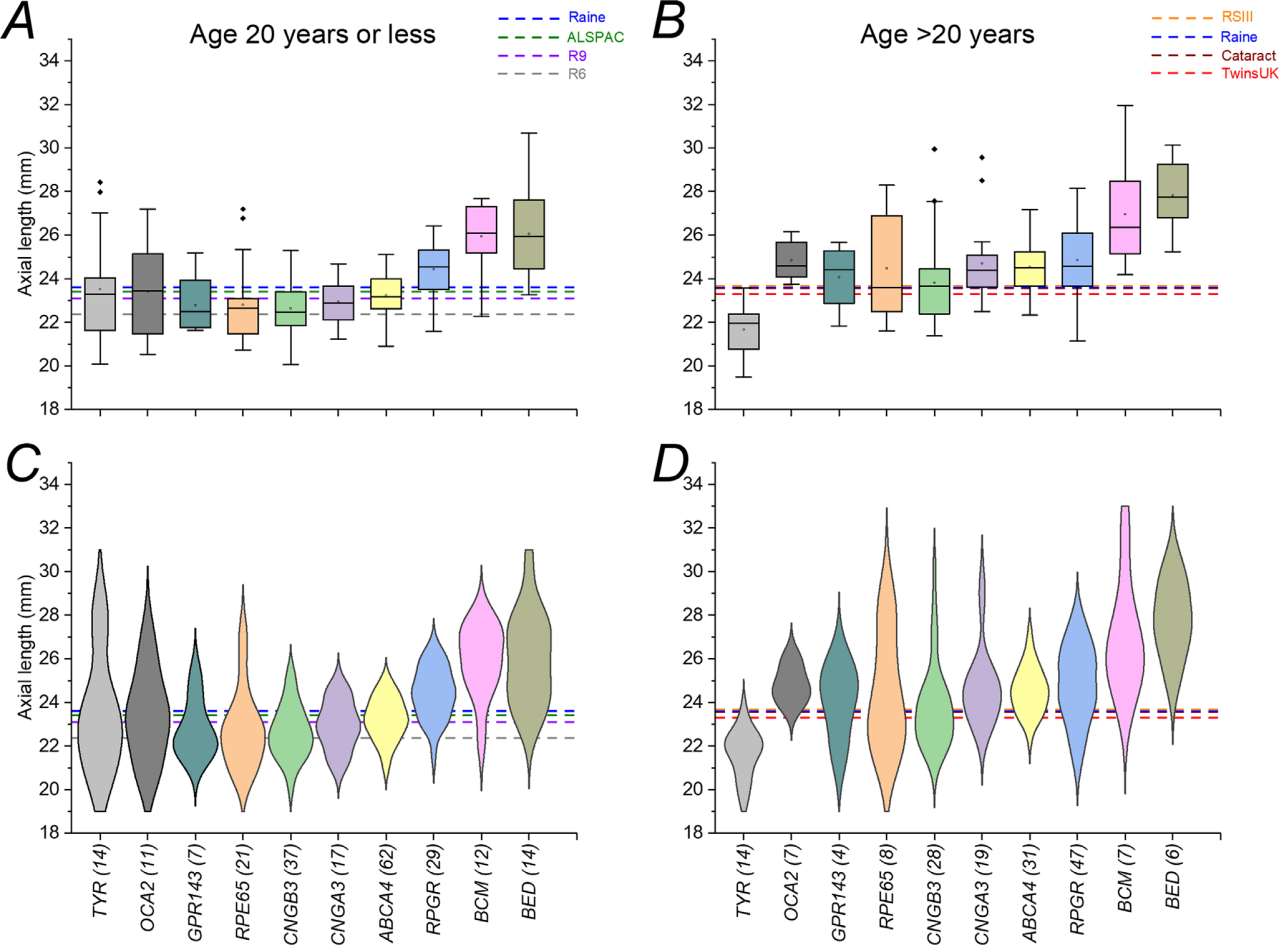
Axial lengths in inherited retinal disease patients divided into younger and older age groups. (**A**, **B**) Boxplots (*open circles* denote means; *filled diamonds* denote outliers). (**C**, **D**) Violin plots. The *left-hand* panels include patients up to 20 years old; the *right-hand* panels include patients older than 20 years. *Horizontal dashed lines* show mean ALs for relevant healthy cohorts. Numbers of patients included in each group are shown in *brackets* for each condition.

As AL in healthy participants has generally stabilized by the age of 20, we divided each IRD cohort into two groups; the younger group included individuals up to 20 years of age, and the older group included individuals over 20 years of age. Figure 5 displays the two groups in the same manner as in Figure 1. Younger and older patients are displayed in the left and right panels, respectively. The horizontal dashed lines show mean ALs from the healthy comparison cohorts: means for younger control cohorts are displayed in the left panels, and means for older control cohorts are displayed in the right panel. The mean age of the Raine Study was at the cut-off, so the mean AL is displayed for comparison in both groups. Broadly, similar trends are seen for both age groups, as mean ALs for BEM, BCM, and *RPGR*-associated disease are higher than in the control cohorts in younger and older patients. There are also some interesting differences. Average ALs in *TYR*- and *OCA2*-associated albinism and in *GPR143*-associated ocular albinism are similar to those of the reference cohorts in younger patients. In older patients, however, average ALs are longer compared with reference cohorts for *OCA2* and *GPR143* but shorter for *TYR*.

### Logistic Regression Model Comparison

Of the reference cohorts, the Raine Study cohort had the closest average age to the IRD cohort. This cohort was used for formal statistical calculation of odds for long and short eyes. The odds of having an AL ≤ 22 mm or AL ≥ 26 mm compared to the Raine Study dataset were examined using adjusted Firth's logistic regression models (Table 3). Significantly increased odds of a longer AL (≥26 mm) were observed for BCM and BED and also (but to a lesser extent) for *TYR*- and *OCA2*-associated albinism, as well as for *RPE65*- and *RPGR-*associated disease. The highest odds of an AL ≥ 26 mm were seen for BCM (adjusted OR, 51.67; *P* < 0.001). Significant odds for a short AL were seen for *GPR143*-associated ocular albinism (adjusted OR, 56.20; *P* < 0.001), *TYR*-associated albinism (adjusted OR, 34.19; *P* < 0.001), and *RPE65-*associated disease (adjusted OR, 12.52; *P* < 0.01).

**Table 3.** Adjusted ORs for AL ≤ 22 mm and AL ≥ 26 mm for Selected Inherited Retinal Diseases Compared With a Reference Cohort (Raine Study)

		AL ≤ 22 mm		AL ≥ 26 mm	
Gene/Condition	*n*	Adjusted OR (95% CI)	*P*	Adjusted OR (95% CI)	*P*
*ABCA4*	82	0.18 (<0.01–5.92)	0.334	2.15 (0.64–7.24)	0.215
BCM	18	4.58 (0.35–60.43)	0.248	51.67^*^ (18.58–143.67)	<0.001
BED	15	4.13 (0.23–73.20)	0.333	48.57^*^ (16.27 -145.06)	<0.001
*CNGA3*	28	4.10 (0.72–23.18)	0.111	2.83 (0.42–19.02)	0.285
*CNGB3*	43	1.39 (0.10–19.01)	0.801	1.42 (0.18–10.94)	0.735
*RPE65*	25	12.52^*^ (3.90–40.21)	<0.001	6.66^*^ (1.82–24.32)	0.004
*RPGR*	60	4.56 (0.85–24.47)	0.077	4.88^*^ (1.74–13.72)	0.003
*TYR*	25	34.19^*^ (13.20–88.53)	<0.001	10.13^*^ (3.06–33.53)	<0.001
*OCA2*	17	0.96 (0.01–171.63)	0.988	6.12^*^ (1.18–31.60)	0.031
*GPR143*	10	56.20^*^ (13.05–241.92)	<0.001	2.66 (0.18–38.21)	0.476

Firth's logistic regression models were constructed with adjustment for age and sex, related individuals excluded, and models only inclusive of males for BCM, BED, *RPGR*, and *GPR143*.

* Adjusted odds ratios with *P* < 0.05.

### Subgroup Analysis in *ABCA4*-Associated Disease

The most frequent IRD in our dataset was *ABCA4*-associated disease. Participants had a mean AL of 23.67 mm (range, 20.91–27.18), with the majority developing visual symptoms before the age of 20 (81% of the 85 patients in whom this was recorded). Although some individuals in this group evidently had significant axial myopia, we found no statistically significant increased risk for an AL over 26 mm.

Individuals with *ABCA4*-associated disease can be further subdivided into one of three functional phenotypes, based on electroretinography (ERG). There is pattern ERG evidence of macular dysfunction in all three groups with full-field ERGs that are normal (group 1), show evidence of generalized cone dysfunction (group 2), or show generalized cone and rod dysfunction (group 3).^26^ These groups appear to have prognostic significance.^28^ We were keen to explore if AL varied among these groups, given the different effects on retinal function. In a linear regression model of unrelated individuals, adjusted for age and sex, we found no significant association between AL and ERG group (*P* = 0.102). Comparing mean ALs (adjusted for age and sex) among the three groups in an ANOVA test—group 1 (*n* = 50, mean AL = 23.94), group 2 (*n* = 8, mean AL = 23.84), group 3 (*n* = 21, mean AL = 23.02)—we found no statistically significant difference (*P* = 0.235). We additionally found no statistical difference when group 2 and 3 individuals were pooled together and compared to group 1 individuals (*P* = 0.119).

### Subgroup Analysis in *RPGR*-Associated Disease

In individuals with *RPGR*-associated disease, we identified five with a cone–rod dystrophy phenotype but the majority (*n* = 71) had a predominant rod–cone phenotype. Individuals with cone–rod dystrophy had longer ALs (mean ± SD, 27.11 ± 1.07 mm) than those with a rod–cone dystrophy (mean ± SD, 24.52 ± 1.45 mm) in a simple *t*-test (*P* = 0.003). In a linear regression model for mean AL adjusted for age and sex, the presence of a cone–rod dystrophy was a significant determinant for a longer AL (β = 2.36, *P* = 0.002). However, if the individuals with a cone-rod dystrophy were excluded, there was still a significant increased odds of longer AL with *RPGR*-associated disease when compared to the reference cohort (adjusted O*R* = 4.01; 95% CI, 1.31–12.29; *P* = 0.015).

### Comparison of *CNGA3*- and *CNGB3*-Associated Achromatopsia

We also performed a subanalysis comparing AL between individuals with *CNGA3*- and *CNGB3*-associated ACHM. The latter genotype appeared to be associated with a shorter average AL, with a peak in the density distribution to the left of that for *CNGA3* (Fig. 2). However, in a simple *t*-test the mean ALs between the two groups *CNGA3* (*n* = 28; mean AL ± SD, 24.03 ± 1.81 mm) and *CNGB3* (*n* = 43; mean AL ± SD, 23.40 ± 1.80 mm) were not significantly different (*P* = 0.153). Similarly, in a linear regression model for mean AL adjusted for age and sex, the genotype *CNGA3* versus *CNGB3* was not a significant determinant (*P* = 0.230).

### Comparison of *OCA2*-, *TYR*-, and *GPR143*-Associated Albinism

We examined data for the three albinism-related genes in our cohort. *TYR*- and *GPR143*-associated albinism was identified as having a higher risk of shorter ALs compared to the reference population. Also, albeit with a lower effect size, both *TYR*- and *OCA2*-associated albinism had significantly higher odds of longer ALs. There was no significant difference in mean AL between the *TYR* and *GPR143* genotypes in a simple *t*-test (*P* = 0.282) or in a linear regression model of only males adjusted for age (*P* = 0.608). The mean AL in individuals with *TYR-*associated albinism (*n* = 25; mean AL ± SD, 22.55 ± 2.30 mm) was significantly shorter than for those with *OCA2-*associated albinism (*n* = 17; mean AL ± SD, 23.99 ± 1.85 mm) in a simple *t*-test (*P* = 0.038). However, in a linear regression for mean AL adjusted for age and sex, the difference between *OCA2* and *TYR* was not significant (*P* = 0.060). Finally, in both a simple *t*-test and linear regression adjusted again for age and sex, the genotype *GPR143* versus *OCA2* was not a significant determinant of AL (*P* = 0.411 and *P* = 0.260, respectively).

## Discussion

In this study, we examined axial lengths in a range of IRDs and compared these AL distributions with those of healthy cohorts. Conditions for which we had more than 10 individuals were those associated with *TYR*, *OCA2*, *GPR143*, *ABCA4*, *CNGA3*, *CNGB3*, *RPGR*, *RPE65*, BCM, and BED. We found that, compared with healthy individuals, AL distributions were broader than in healthy cohorts, indicating likely disruption of mechanisms of emmetropization. Individuals with BCM, BED, *RPGR*-associated disease, and *OCA2*-associated oculocutaneous albinism showed significantly increased odds for having an AL of 26 mm or greater. Individuals with *TYR*-associated oculocutaneous albinism and those with *RPE65*-associated disease showed significantly increased odds for longer ALs (≥26 mm) and also for shorter ALs (≤22 mm). Individuals with *GPR143*-associated ocular albinism had significantly increased odds for shorter (≤22 mm) eyes.

We also found intriguing differences in the albinism groups when divided by age. Younger patients had mean ALs similar to healthy cohorts. Older patients (older than 20 years) showed a divergence (shorter mean lengths in *TYR* and longer ALs in *OCA2*- and *GPR143*-associated disease compared with healthy cohorts), although small numbers in the subgroups make these findings somewhat tentative.

*ABCA4* is the gene most frequently associated with monogenic retinal disease. Bi-allelic pathogenic variants give rise to Stargardt disease, with onset in early childhood or early adulthood; patients can exhibit a macular dystrophy or a cone or cone–rod dystrophy. The gene is expressed in photoreceptors, and the encoded protein functions in the visual cycle. Disturbance of function leads to build up of lipofuscin-related material primarily in the retinal pigment epithelium (RPE) and degeneration of the outer retina. Although discrete hyperautofluorescent flecks are often seen on retinal imaging, quantitative autofluorescence studies have shown a much more generalized accumulation of autofluorescent material, with some correlation with severity of the pathogenic variants.^10^^,^^29^ Our finding that patients’ ALs did not differ significantly from our reference cohort suggests that disruption of protein function does not predispose to abnormally long or short eye growth. The distribution of ALs was also similar to those of the reference cohorts, and we found no significant difference in AL among the ERG subtypes (groups 1, 2, and 3, broadly representing macular, cone, and cone–rod dystrophy groupings, respectively). Interestingly, a report in 1982 found that patients with Stargardt disease appeared to have greater myopic spherical equivalent^30^; however, the control group for this study was taken from measurements made in 1957,^31^ so the results may not have been comparable if prevalence of myopia had increased over the intervening decades.

Bi-allelic pathogenic variants in *RPE65* give rise to an early-onset retinal dystrophy, often from birth, for which gene therapy is now available and licensed in many countries. The gene is expressed in the RPE, and the encoded protein is critical to the visual cycle (more so than *ABCA4*), such that retinoid recycling is severely impaired and patients show very low autofluorescence levels on retinal imaging.^11^ Patients have early-onset severe impairment, particularly in lower light levels, and the peripheral retina degenerates very early, with subsequent degeneration of the macula (whereas *ABCA4*-associated disease is later onset and macula first). Here, we found that ALs were more widely distributed compared with healthy cohorts, with greater odds of a shorter and a longer AL; this might be consistent with a general impairment of mechanisms of emmetropization.

*RPGR*-associated disease accounts for the majority of X-linked retinitis pigmentosa (RP) cases (patients can also display a later onset macula, cone, or cone–rod dystrophy). The protein localizes to photoreceptor connecting cilia,^32^ and patients with RP are usually more severely affected than those with autosomal forms of RP. Myopia is a known association, and our study confirmed that patients had, on average, longer ALs, with greater odds for longer ALs. Interestingly, patients with the cone–rod phenotype (who may present later in life) had significantly longer ALs than those with a rod–cone phenotype, although we had few participants with the cone–rod phenotype.

Variants in *CNGA3* and *CNGB3* are the most frequent causes of ACHM.^33^^–^^35^ These genes encode subunits of the cyclic nucleotide–gated channel in cone photoreceptor outer segments. These channels close in response to the light-induced depletion of cyclic guanosine monophosphate (cGMP), causing the photoreceptor to hyperpolarize in response to light. Patients have an absence of cone function from birth. We did not find increased odds of longer or shorter eyes in patients with *CNGA3* or *CNGB3* variants, suggesting that eye growth may not be significantly affected. However, analysis of the distributions (Fig. 2) does show the existence of possibly much smaller peaks at longer ALs, suggesting disruption of emmetropization in some people. It is interesting that our patients with *PDE6C*-associated ACHM showed a longer average AL, suggestive of higher myopia. The number of these patients was small, but it is possible that different mechanisms of ACHM have different effects on eye growth. In *PDE6C*-associated disease, cGMP might be expected to remain high, meaning that the cone photoreceptor cyclic nucleotide–gated channels presumably remain open in light (and so the cones are depolarized) in contrast to *CNGA3*- and *CNGB3*-associated disease, where the channels are likely not to be functional (and so the cones might remain hyperpolarized). Also, relative preservation of S-cone function has been reported in *PDE6C*-associated ACHM,^8^ and it is possible that this somehow predisposes to myopia given the association we found between BCM and increased AL.

BCM is also a cone dysfunction syndrome, but here the S-cones remain functional, with loss of L- and M-cone function. These patients showed significantly increased odds of having longer eyes. It is possible that the L- and M-cones again remain depolarized in light (similar to *PDE6C*-associated disease, as hypothesized above), although their structural integrity might also be impaired. Also, unlike L- and M-cones, S-cones are relatively sparse, with differing onward connectivity,^36^ forming opponent pathways with L- and M-cones (the latter input will likely be absent in BCM), and this could be contributory. In BED, patients are dichromats, with pathogenic variants affecting the L- or M-cone opsin and possibly a skewed L:M cone ratio, giving rise to generalized cone dysfunction.^37^^,^^38^ We found that these patients also had greater odds of a longer AL. Of the conditions studied, BCM and BED were associated with the greatest odds of a longer AL compared with the reference cohort, with odds ratios of over >30 and >80, respectively.

Our findings in patients with different genetic causes of albinism showed intriguing and contrasting patterns. Although both *TYR* and *OCA2* are associated with autosomal recessive OCA, the AL distribution in *OCA2*-associated disease showed a peak at longer ALs, whereas the distribution in *TYR*-associated disease showed a peak at shorter lengths. In *GPR143*-associated X-linked ocular albinism, the major peak was also at shorter lengths. For all three genes, there were clear secondary peaks also. Compared to the reference cohort, individuals with *OCA2*-associated albinism had significantly increased odds for longer eyes; those with *TYR*-associated albinism had increased odds for both longer and shorter eyes (but the odds ratio for shorter eyes was greater); patients with *GPR143*-associated disease had the greatest odds ratio for shorter eyes (56.20) of all the conditions studied.

There have been several genome-wide association studies (GWASs) for refractive error, with some more specifically exploring associations with AL or corneal curvature.^39^^,^^40^ In the largest GWAS for refractive error published to date,^41^ a large number of loci were associated with refractive error. Of the six autosomally inherited conditions considered in the present study, three of the genes (as implicated by single nucleotide polymorphisms [SNPSs]) reached genome-wide significance in that meta-analysis:^41^ *ABCA4* (best SNP rs11165052, *P* = 3.20 × 10^−10^), *CNGB3* (best SNP rs13268738, *P* = 1.01 × 10^−14^), and *OCA2* (best SNP rs79406658, *P* = 1.37 × 10^−15^). Both BED and BCM relate to the opsin gene cluster on Xq28. In the large refractive error meta-analysis,^41^ two SNPs on Xq28 reached genome-wide significance: rs4828621 (closest gene *MAMLD1*) and rs180874045 (closest gene *TKTL1*). The latter SNP is less than 50,000 base pairs from the L- and M-cone opsin cluster. The opsin gene cluster has relevance to myopia^42^ and has been identified as having the locus for X-linked high-grade myopia (MYP1).^37^^,^^43^ Some studies have highlighted the potential relevance of the variation in L- and M-cone ratios to refractive error in healthy individuals,^44^^,^^45^ with animal studies supporting an association with axial length and refraction.^46^ We did not examine the *OPN1LW* and *OPN1MW* genes in the non-BED/BCM cohorts, although future studies might incorporate such analyses to examine a modifying role of these genes

There were some limitations of our study. We had relatively small numbers of participants within some IRD groups, limiting the power of some of our comparisons and precluding a comparison within each condition (for example, to examine if different pathogenic variants were associated with different ALs). As AL changes nonlinearly with age (as shown in the reference cohorts in Fig. 3A), differences in age distributions between patients and reference cohorts can confound comparisons. We sought to mitigate this by including a number of reference cohorts with different average ages to give an idea of the relation with age but making formal statistical comparison with the cohort with the most similar average age. Also, we did not have information on known myopia risk factors and so could not adjust for these. In addition, refractive error data were not available for this study; future studies incorporating both axial length and refraction will be informative. The majority of axial lengths in the present study were measured with the same type of biometer (IOLMaster). In the TwinsUK cohort, two other biometers (see Methods) were used in some participants, representing another potential limitation. However, previous studies have shown very close agreement between these devices, with any differences being small.^47^^,^^48^ The range of conditions studied was also limited to those in which natural history studies were being undertaken, leading to possible ascertainment bias, and the full spectrum of IRD was not included; for example, X-linked and autosomal recessive forms of complete congenital stationary night blindness are known to be associated with high myopia^49^^,^^50^ but were not included here.

To the best of our knowledge, this is the largest study of AL in IRD, in spite of the limited number of disorders investigated. Of the conditions studied, the majority (*ABCA4*, *CNGA3*, *CNGB3*, *RPGR*, and *RPE65*) are among the top 20 genes associated with IRD^6^ and are also the subject of novel treatment trials, including gene therapy (with *RPE65*-associated disease having an available licensed treatment). We have described AL distributions in these conditions and compared these with healthy cohorts. Such information can inform the differential diagnosis when seeing patients with undiagnosed IRD and potentially shed light on mechanisms of emmetropization, of particular relevance because of the increasing prevalence of myopia.

Beyond emmetropization, differences in ALs among the various conditions have implications for retinal imaging biomarkers. Differences in AL affect the retinal magnification factor of the eye and, when not corrected for, can result in significant errors in quantitative measurements, especially when they deviate from normal values.^51^^,^^52^ Caution is therefore required when comparing imaging results across IRDs when the effect of AL on retinal magnification has not been taken into account.

Our findings also show that broad statements about predominant cone or rod dysfunction predisposing to myopia are of limited accuracy: we found that different cone dysfunction syndromes were associated with varying effects on eye growth and that longer ALs could be a feature of disease associated with a particular gene (*RPGR*) whether the rods or the cones degenerated first, although here an interesting difference was found between the subgroups. An apparently single clinical syndrome could yield different AL distributions depending on the particular genetic cause, illustrated by our findings in albinism. A future study measuring AL in a broader spectrum of IRDs, and serially over time, will likely yield further insights.
